# Enhanced Recovery After Surgery (ERAS) in Pancreatic Surgery: The Surgeon’s Point of View

**DOI:** 10.3390/jcm13206205

**Published:** 2024-10-18

**Authors:** Fabio Longo, Edoardo Panza, Lorenzo Rocca, Beatrice Biffoni, Chiara Lucinato, Marco Cintoni, Maria Cristina Mele, Valerio Papa, Claudio Fiorillo, Giuseppe Quero, Davide De Sio, Roberta Menghi, Sergio Alfieri, Lodovica Langellotti

**Affiliations:** 1 Digestive Surgery Unit, Fondazione Policlinico Universitario A. Gemelli IRCCS, Largo A. Gemelli 8, 00168 Rome, Italy; edoardo.panza01@icatt.it (E.P.); lorenzo.rocca01@icatt.it (L.R.); beatrice.biffoni01@icatt.it (B.B.); chiara.lucinato01@icatt.it (C.L.); valerio.papa@policlinicogemelli.it (V.P.); claudio.fiorillo@policlinicogemelli.it (C.F.); giuseppe.quero@policlinicogemelli.it (G.Q.); davide.desio01@gmail.com (D.D.S.); roberta.menghi@policlinicogemelli.it (R.M.); sergio.alfieri@policlinicogemelli.it (S.A.); lodovica.langellotti01@icatt.it (L.L.); 2 UOC Nutrizione Clinica, Dipartimento di Scienze Mediche e Chirurgiche, Fondazione Policlinico Universitario A. Gemelli IRCCS, Largo A. Gemelli 8, 00168 Rome, Italy; marco.cintoni@policlinicogemelli.it (M.C.); mariacristina.mele@policlinicogemelli.it (M.C.M.); 3 Centro di Ricerca e Formazione in Nutrizione Umana, Università Cattolica del Sacro Cuore, 00168 Rome, Italy; 4 Dipartimento di Medicina e Chirurgia Traslazionale, Università Cattolica del Sacro Cuore, Largo Francesco Vito 4, 00168 Roma, Italy

**Keywords:** pancreatic surgery, enhanced recovery after surgery, delayed gastric emptying, pancreatic fistula, biliary drainage, nutrition

## Abstract

Pancreatic surgery is complex and associated with higher rates of morbidity and mortality compared to other abdominal surgeries. Over the past decade, the introduction of new technologies, such as minimally invasive approaches, improvements in multimodal treatments, advancements in anesthesia and perioperative care, and better management of complications, have collectively improved patient outcomes after pancreatic surgery. In particular, the adoption of Enhanced Recovery After Surgery (ERAS) recommendations has reduced hospital stays and improved recovery times, as well as post-operative outcomes. The aim of this narrative review is to highlight the surgeon’s perspective on the ERAS program for pancreatic surgery, with a focus on its potential advantages for perioperative functional recovery outcomes.

## 1. Introduction

Pancreatic cancer is one of the most detrimental tumors, characterized by a dismal prognosis [1]. Many patients present with single or multiple metastases at the time of diagnosis. However, the efficacy of new chemotherapy protocols is increasing the number of patients eligible for surgery [1]. Despite these advancements, pancreatic surgery remains a high-risk procedure associated with significant morbidity and mortality rates [1]. Common complications include postoperative pancreatic fistula (POPF), delayed gastric emptying (DGE), postoperative hemorrhage, and intra-abdominal infections, all of which adversely affect patients’ oncological outcomes and quality of life [1]. In recent decades, improvements in surgical techniques, driven by the centralization of pancreatic surgery in high-volume specialized centers, combined with advancements in perioperative management, have reduced mortality rates [2]. However, post-operative complication rates remain high, leading to considerable morbidity and discomfort. In 2012, the “Enhanced Recovery After Surgery (ERAS) study group” developed a series of evidence-based recommendations for the pre-operative, intra-operative and post-operative management of patients, aimed at reducing perioperative complications, improving outcomes, and decreasing costs and hospital stays [2]. Since the publication of the ERAS guidelines, their safety and effectiveness in abdominal surgery have been evaluated through several randomized controlled trials, meta-analyses, and retrospective studies [3,4,5,6,7,8,9,10,11]. Clinical trials on pancreatic surgery [12,13,14,15,16,17,18] are summarized in Supplementary Table S1. The objective of this review is to offer a comprehensive analysis of the ERAS guidelines in the context of pancreatic surgery, with particular emphasis on their clinical benefits and impact on perioperative outcomes.

## 2. Materials and Methods

A narrative review of the current literature was conducted using PubMed and Scopus databases. To achieve this objective, the following key words related to ERAS recommendations for pancreatic surgery were searched: (‘pancreatic surgery’ AND ‘pre-operative biliary drainage’), (‘pancreatic surgery’ AND ‘peri-anastomotic drainage’), (‘pancreatic surgery’ AND ‘somatostatin analogues’), (‘pancreatic surgery’ AND ‘postoperative fistula’), (‘pancreatic surgery’ AND ‘delayed gastric emptying’), (‘pancreatic surgery’ AND ‘nasogastric intubation’), (‘pancreatic surgery’ AND ‘nutrition’), and (‘pancreatic surgery’ AND ‘post-operative analgesia’). The inclusion criteria were limited to studies published in English, involving human subjects, and consisting of clinical trials, meta-analyses, systematic reviews, or observational and comparative studies.

## 3. Discussion

### 3.1. Preoperative Biliary Drainage

Pancreatic ductal adenocarcinoma (PDAC) is the fourth leading cause of cancer-related death, with a poor prognosis median 5-years survival of 8.1%, median 10-years overall survival of 3% [19]. Only 10–20% of patients affected by PDAC, may be eligible for surgical resection [19]. The most common symptom of pancreatic head neoplasms at the time of diagnosis is obstructive jaundice, which may be associated with coagulopathy, malnutrition, malabsorption and significant patient discomfort [19,20].

Although biliary drainage is the treatment of choice for jaundice, it is an invasive procedure with the potential for periprocedural complications. Several studies [21,22,23] suggest that preoperative biliary drainage may reduce postoperative morbidity, while other studies [24,25,26] indicate that it may increase the risk of postoperative complications, particularly infectious ones. As a result, no clear consensus has been reached on this issue [21,22,23,24,25,26,27]. Consequently, the decision to perform preoperative biliary drainage, aimed at optimizing the patient’s clinical condition prior to surgery, remains a controversial topic in the literature.

#### 3.1.1. Who to Drain?

According to ERAS recommendations [2], there is strong evidence that preoperative biliary drainage is not always necessary, as it appears to increase postoperative complications without offering survival benefits. However, preoperative biliary drainage is recommended for patients presenting with jaundice and elevated bilirubin levels (>15 mg/dL), those undergoing neoadjuvant chemotherapy, or those affected by recurrent cholangitis.

These recommendations are in alignment with the current According to National Comprehensive Cancer Network (NCNN) Guidelines (2024 version) [19] which do not advocate routine preoperative biliary drainage before surgery. However, drainage may be warranted in cases of cholangitis, severe jaundice, pruritus, or when surgery is delayed due to neoadjuvant chemotherapy or other clinical factors. Similarly, the European Society for Medical Oncology (ESMO) guidelines [28] recommend preoperative biliary drainage only in patients with serum bilirubin levels exceeding 250 mmol/L, in those scheduled for neoadjuvant chemotherapy, or when surgical intervention is postponed for more than two weeks. Notably, preoperative drainage is associated with a higher complication rate and is therefore deemed unnecessary in patients with bilirubin levels below 250 mmol/L.

Current literature further corroborates that routine biliary drainage for pancreatic cancer-associated jaundice is not indicated [21,22,23,24,25,26,27].

A multicenter retrospective study [21] including 289 patients found no statistically significant difference between the drain and no-drain groups in terms of postoperative mortality and complications, namely POPF, biliary fistula, intestinal fistula, incision complication, DGE, organ-related complications, and postoperative hospital stay. However, a subgroup analysis of patients with elevated bilirubin levels revealed that severe complications occurred significantly more frequently in the no-drain cohort (*p* = 0.028), suggesting that bilirubin levels higher than 300 mmol/L may be a predictor of poor surgical outcomes.

Two retrospective studies [24,26] reported a significantly higher morbidity rate in patients with prolonged biliary drainage. Wang et al. [24] demonstrated a significantly higher overall rate of postoperative complications among patients who underwent biliary drainage for more than two weeks (χ^2^ = 6.102, *p* = 0.013), with an even greater incidence of severe complications associated with prolonged drainage (χ^2^ = 4.673, *p* = 0.03). A 2013 meta-analysis [22] involving 3532 patients found a lower rate of major complications in the drain group (10.40%; 95% CI: 9.96–10.83) compared to the no-drain group (15.56%; 95% CI: 15.06–16.05), although no differences were observed in postoperative mortality or length of stay. Conversely, another meta-analysis [25] identified increased morbidity rates in the drain group, with no associated survival benefit.

These findings suggest that preoperative biliary drainage should not be performed routinely, but rather reserved for specific patient subgroups as previously discussed.

#### 3.1.2. How to Drain?

Preoperative management of jaundice can be achieved through either percutaneous biliary drainage or endoscopic stent placement, with endoscopic stents available in both plastic and metallic forms. According to ERAS guidelines [2], no strong recommendation exists regarding the superiority of one treatment over the other. Percutaneous biliary drainage appears to offer no significant clinical advantage over the endoscopic one and is generally less well tolerated. With respect to endoscopic stents, ERAS guidelines [2] indicate no significant differences in postoperative complications between the use of metal and plastic stents.

According to the NCCN guidelines [19], the optimal approach for biliary drainage is endoscopic stent placement, with a preference for metallic stents. Plastic stents should be reserved for palliative care in patients with a poor prognosis (less than three months). If endoscopic drainage is technically unfeasible, percutaneous drainage should be considered. Recent literature [29,30] suggests that metal stents are safer compared to plastic stents, primarily due to their lower occlusion rates.

A recent meta-analysis [29] compared plastic and metallic stent placement in terms of efficacy and safety. Metallic stents were associated with a significantly lower re-intervention rate compared to plastic stents (OR, 0.06; 95% CI 0.03–0.15; *p* < 0.001), due to lower rates of stent migration and obstruction. However, both strategies were comparable in terms of surgical complication rates and postoperative mortality. Metallic stents also demonstrated a longer patency rate after three months (70–100% versus 30–45%) and reduced the need for re-interventions.

Another recent meta-analysis [30] comparing percutaneous and endoscopic procedures found no significant differences in terms of severe postoperative complications, postoperative mortality, or wound infection rates between the two approaches. However, percutaneous drainage was associated with a lower incidence of periprocedural and postoperative complications (OR: 0.7, 95% CI: 0.52–0.94; *p* = 0.02, and OR: 0.44, 95% CI: 0.23–0.84; *p* = 0.01, respectively).

In conclusion, the decision to place preoperative biliary drainage in patients with pancreatic cancer should be individualized, taking into account factors such as the patient’s clinical condition, the severity of jaundice, the presence of cholangitis, and the need to delay surgery for chemotherapy. A multidisciplinary approach involving surgeons, gastroenterologists, and oncologists is essential to optimize patient outcomes. The current trend favors a selective, rather than routine, use of percutaneous biliary drainage, reserving it for cases where clear clinical indications exist. Elevated preoperative bilirubin levels necessitate biliary drainage, as patients with bilirubin levels >15 mg/dL who underwent drainage had a significantly lower rate of severe postoperative complications compared to those who proceeded directly to surgery. However, this benefit was not observed in patients with bilirubin levels below 15 mg/dL.

Except in cases where it is not technically feasible, endoscopic drainage is generally preferred over percutaneous drainage, as it offers comparable peri-procedural complication rates and postoperative outcomes while causing less patient discomfort. Additionally, metallic stents are favored, with plastic stents reserved for patients with a life expectancy of less than three months or for those undergoing upfront surgery.

### 3.2. POPF

POPF is defined as the detection of fluid output from surgically placed abdominal drains containing amylase levels greater than three times the upper limit of normal serum values [31,32]. A clinical grading system categorizing POPF into three levels—‘biochemical leak’, grade B, and grade C—was introduced based on the severity of complications [32]. POPF occurs in approximately 19% of patients undergoing pancreatic surgery and remains the leading cause of morbidity following pancreatic resection [31,32,33]. Several risk factors significantly increase the likelihood of POPF, including male sex, body mass index > 25 kg/m^2^, pancreatic duct diameter < 3 mm, soft pancreatic texture, high-risk pathology (anything other than pancreatic cancer or pancreatitis), and increased intraoperative blood loss [33].

#### 3.2.1. Peri-Anastomotic Drainage

Intra-abdominal drain placement during pancreatic surgery has been demonstrated to reduce morbidity and mortality in patients who develop POPF [34]. According to ERAS recommendations [2], intraoperative drain placement with early removal (within 72 h) is advised for patients at low risk of POPF, identified by drainage amylase levels < 5000 U/L on post-operative day 1. On the counterpart, recent literature [34,35,36,37,38,39] does not provide clear evidence supporting a ‘no-drain’ approach in pancreatic surgery.

Early drain removal has become increasingly common over time. Several prospective clinical trials [33,35,36,37,38] have demonstrated that early drain removal is associated with significant reductions in the incidence of clinically relevant POPF, as well as shorter postoperative hospital stays, leading to faster recovery, in line with the principles of Enhanced Recovery After Surgery (ERAS) [33,34,35,36,37]. Bassi et al. [36] further demonstrated that early drain removal was linked to a reduced rate of POPF (*p* = 0.001), and lower rates of abdominal and pulmonary complications (*p* = 0.002 and 0.007, respectively). Therefore, in patients at low risk for POPF, intra-abdominal drains can be safely removed by postoperative day 3, as prolonged drainage may be associated with a higher incidence of postoperative complications, extended hospital stays, and increased costs.

A prospective study [38] on 104 patients identified drain removal after postoperative day 4 as an independent risk factor for abdominal complications, such as postoperative infections. In this study, patients were assigned to group 1 (drain removed on postoperative 8) or group 2 (drain removed on postoperative day 4), and postoperative complications were compared between the two groups. The incidence of POPF was significantly lower in group 2 (3.6%) compared to group 1 (23%) (*p* = 0.0038). Additionally, the rate of intra-abdominal infections, including abscesses and infected collections, was significantly reduced in group 2 (7.7%) compared with group 1 (38%) (*p* = 0.0003).

On the other hand, the complete omission of surgical drainage appears to be associated with a higher complication burden and mortality rate in all cases [38]. However, some authors suggest that the use of drains may increase the risk of clinically relevant POPF, particularly following distal pancreatectomy, indicating that routine drainage may not be advisable in certain pancreatic procedures [39]. In line with this debate, a 2024 Italian survey [34] highlighted the conservative approach of Italian pancreatic surgeons, who continue to favor drainage placement despite recent literature advocating for early removal or even omission.

#### 3.2.2. Somatostatins Prophylaxis

According to ERAS recommendations [2], there is moderate evidence suggesting that the prophylactic administration of somatostatin analogues (SA) may reduce the severity of POPF. However, their routine use is not recommended, as the results of clinical trials have yet to be fully validated. A literature search by the ERAS study group identified 13 randomized controlled trials and 12 systematic reviews on this topic. The largest systematic review [40], which included 21 trials and 2348 patients, reported no significant difference in mortality but found a lower incidence of POPF in patients premedicated with SA (RR: 0.66, 95% CI: 0.55–0.79; *n* = 2206). Although perioperative mortality did not differ significantly between the groups (RR: 0.80; 95% CI: 0.56–1.16; *n* = 2210), the incidence of perioperative complications was significantly lower in patients receiving SA (RR: 0.70, 95% CI: 0.61–0.80; *n* = 1903).

According to NCCN guidelines [19], octreotide does not appear to reduce POPF rates, as evidenced by two prospective, randomized, double-blind, placebo-controlled studies [40,41,42]. Based on these findings, the routine prophylactic use of octreotide cannot be recommended.

Lowy et al. [41] conducted a randomized trial comparing subcutaneous postoperative octreotide administration versus no treatment following pancreatoduodenectomy for malignancy. Demographic and clinical characteristics were similar between the two groups, and surgical techniques were standardized. The rate of clinically relevant POPF was 12% in the octreotide group and 6% in the control group (*p* = 0.23), with perioperative morbidity rates of 30% and 25%, respectively. Similarly, Yeo et al. [42] reported comparable results: pancreatic fistula rates were 9% in the control group and 11% in the octreotide group, overall complication rates were 34% and 40%, and in-hospital mortality rates were 0% and 1%, respectively. The median postoperative length of hospital stay was 9 days in both groups.

In contrast, a single-center, double-blind randomized clinical trial [43] found that pasireotide significantly reduced the rate of POPF and intra-abdominal abscess. Allen et al. [43] demonstrated that the incidence of postoperative complications, such as POPF, intra-abdominal abscess, or anastomotic leak, was significantly lower in patients receiving pasireotide compared to placebo (9% vs. 21%; RR: 0.44, 95% CI: 0.24–0.78; *p* = 0.006).

These findings underscore the need for further validation in future clinical trials.

### 3.3. DGE

Delayed gastric emptying (DGE) is a common complication following pancreatic surgery, with an incidence ranging from 5% to 30%, particularly after pancreatoduodenectomy [44]. Patients who develop DGE experience a significantly reduced quality of life during the first year post-surgery [45]. The International Study Group of Pancreatic Surgery (ISGPS) [45] has established an objective definition of DGE, characterized by the inability to resume a normal diet by the end of the first postoperative week, often necessitating prolonged nasogastric intubation. DGE is classified into three grades (A, B, and C), depending on its severity and impact on clinical outcomes and postoperative management.

Several risk factors contribute to the development of DGE after pancreatic surgery, including surgical trauma, postoperative inflammation, and gastric denervation. Additionally, postoperative abdominal complications such as intra-abdominal fluid collections or abscesses, low preoperative albumin levels, and the use of proton pump inhibitors have been identified as independent risk factors for DGE [46,47,48,49,50,51,52,53,54,55,56,57,58,59].

DGE typically presents with symptoms such as nausea, abdominal pain, bloating, and vomiting, leading to delayed oral feeding, malnutrition, discomfort, and prolonged hospitalization [60]. The management of DGE after pancreatic surgery generally follows a conservative approach [61]. Patients who can tolerate oral intake should consume smaller, more frequent meals and avoid foods that are difficult to digest. Prokinetic medications, which promote gastric emptying, may be prescribed to alleviate symptoms and stimulate peristalsis. In severe cases, where oral intake is inadequate or impossible, enteral feeding may be required to ensure adequate nutrition and maintain mucosal integrity [61]. In certain cases, endoscopic procedures such as pyloric dilation or mechanical dilation of a collapsed bowel may be performed to relieve the obstruction. For rare, persistent, or severe cases of DGE, revision surgery may be considered to correct anatomical or functional issues contributing to the condition [62].

#### 3.3.1. Nasogastric Tube

The intraoperative placement of a nasogastric tube (NGT) has been traditionally performed for decades in order to prevent aspiration pneumonia and post operative ileus, although there is no clear consensus on its routine use after pancreaticoduodenectomy [45]. According to ERAS recommendations [2], the NGT should be removed in the operating room following surgery, and its routine use is not advised. Similarly, the ISGPS [45] recommends removing the NGT as early as possible after pancreatic resection, with some centers opting to remove it at the time of patient extubation.

A recent meta-analysis [11] found that ERAS protocols for pancreaticoduodenectomy were associated with reduced hospital stay, morbidity, and rates of DGE. The time to liquid and solid intake, as well as the first stool passage, was significantly shorter in the ERAS group (mean difference (MD): −3.23 days, 95% CI: −4.62 to −1.85; *p* < 0.001; MD: −3.84 days, 95% CI: −5.09 to −2.60; *p* < 0.001; MD: −1.38 days, 95% CI: −1.82 to −0.94; *p* < 0.001, respectively). Additionally, time to NGT removal was shorter in the ERAS group across all studies (MD: −3.03 days, 95% CI: −4.87 to −1.18; *p* = 0.001). The overall complication rate was also lower in the ERAS cohort (risk difference (RD): −0.04, 95% CI: −0.08 to −0.01; *p* = 0.015), while mortality rates were comparable between the two groups. The duration of hospital stay was significantly shorter in the ERAS group (MD: −2.33 days, 95% CI: −2.98 to −1.69; *p* < 0.001).

The majority of studies concerning ERAS recommendations in pancreatic surgery have been single-center, retrospective, and based on small sample sizes. However, the Impact of the Absence of Nasogastric Decompression After Pancreaticoduodenectomy (IPOD) study [63] was a randomized clinical trial conducted at a high-volume pancreatic surgery university hospital in France. This study demonstrated comparable postoperative complication rates between patients in the NGT group and those in the no-NGT group after pancreatic surgery. In the no-NGT group, the nasogastric tube was removed immediately after surgery, while in the NGT group, it was removed between the third and fifth postoperative days. The primary outcome was the incidence of postoperative complications, and the results confirmed that not placing an NGT is safe after pancreatic surgery. Postoperative complication rates of grade II or higher were similar between the two groups (RR: 0.99; 95% CI: 0.66–1.47; *p* > 0.99).

Furthermore, a meta-analysis [64] highlighted a possible correlation between routine postoperative NGT placement and the incidence of DGE, suggesting that early removal of the NGT not only enhances safety but also reduces the risk of DGE and shortens hospital stay. The studies included in the meta-analysis did not support routine NGT placement, as it was associated with a higher incidence of overall (OR: 2.51, 95% CI: 1.12–5.63; *p* = 0.03) and more severe DGE (OR: 3.64, 95% CI: 1.83–7.25; *p* < 0.01), a higher rate of Clavien–Dindo grade II or higher complications (OR: 3.12, 95% CI: 1.05–9.28; *p* = 0.04), and prolonged hospital stay (MD: 2.67 days, 95% CI: 0.60–4.75; *p* = 0.02).

#### 3.3.2. Surgical Techniques

The influence of different surgical techniques and their role in decreasing the incidence of DGE has been extensively investigated. An active debate continues regarding the optimal surgical gastrointestinal reconstruction techniques, including antecolic (AC) versus transmesocolic (TMC) gastrojejunostomy reconstruction, pancreatojejunostomy versus pancreatogastrostomy, and pylorus-preserving pancreaticoduodenectomy (PPP) versus the Whipple procedure (WP). According to the ERAS study group [2], several meta-analyses [65,66,67] have evaluated the relationship between surgical techniques and DGE incidence, finding no significant differences in DGE rates.

Specifically, a meta-analysis [65] of eight randomized clinical trials investigated the impact of AC versus TMC reconstruction and found no significant differences between the two techniques regarding the incidence of DGE, morbidity, mortality, quality of life, or length of hospital stay.

Two additional meta-analyses, conducted by Ma et al. [66] and Lei et al. [67], compared pancreatogastrostomy and pancreatojejunostomy anastomoses in terms of complications such as the incidence of POPF, intra-abdominal fluid collections, DGE, hemorrhage, re-intervention rates, morbidity, and mortality. Ma et al. [66] reported that pancreatogastrostomy was associated with significantly lower rates of POPF and intra-abdominal fluid collections (OR: 0.53, 95% CI: 0.37–0.74; *p* < 0.001, and OR: 0.48, 95% CI: 0.30–0.76; *p* < 0.01), but found no superiority for reducing DGE.

Similarly, Lei et al. [67] demonstrated the potential superiority of pancreatogastrostomy in terms of reducing the incidence of POPF (*p* = 0.01) and biliary fistula (*p* = 0.02) but found no significant differences in DGE rates between the two reconstruction techniques.

Significant interest surrounds the comparison between PPP and WP in terms of DGE incidence. Two prospective randomized trials [68,69] and two meta-analyses [70,71,72] compared PPP and WP, but found no correlation between the surgical technique and the incidence of DGE.

On the counterpart, Hackert et al. [73] reported that PPP should remain the standard of care, as pylorus resection does not reduce the incidence or severity of DGE within 30 days after surgery.

**Figure 1:**
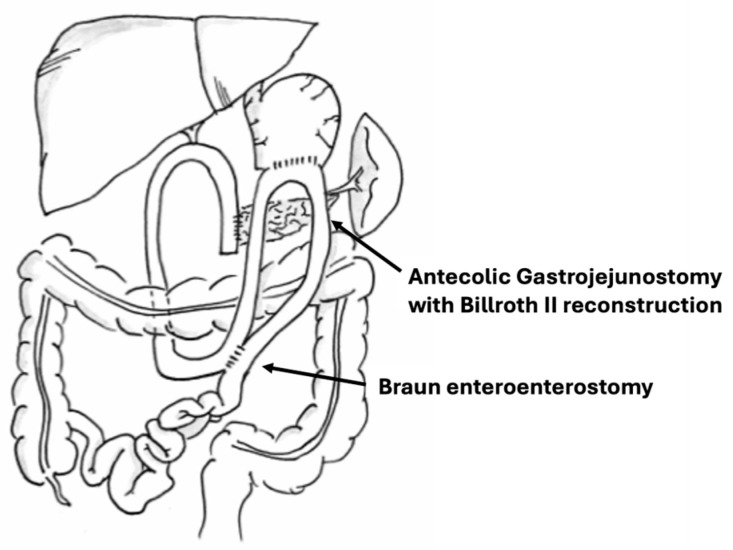
Whipple procedure (no pylorus preservation) with an antecolic gastrojejunostomy and Braun entero-enterostomy.

A recent meta-analysis [70], which included 24 randomized controlled trials comparing various gastric resection techniques (WP, PPP), anastomotic routes (AC, TMC), configurations (loop gastroenterostomy/Billroth II, Roux-en-Y), and the use of enteroenterostomy (Braun), found that a WP, AC, Billroth II reconstruction with Braun enteroenterostomy was associated with the lowest rates of DGE (Figure 1).

Furthermore, in patients who underwent the WP, AC gastrojejunostomy was shown to be superior to TMC gastrojejunostomy in reducing the incidence of DGE. Antecolic placement of the duodenojejunostomy also appears to be advantageous in patients undergoing PPP [69,71,72,74,75,76].

Moreover, side-to-side gastrojejunostomy has demonstrated clear advantages compared to end-to-side reconstruction [77]. Recently, increasing attention has been given to the configuration of the gastrojejunostomy, specifically the impact of the flow angle at the anastomosis between the stomach and jejunum. A reduced flow angle, resulting from a vertical anastomosis of the jejunal limb to the stomach, has been reported to significantly improve food passage by gravity, potentially preventing the development of DGE. The advantages of this technical variation were first demonstrated after distal gastrectomy with a Roux-en-Y reconstruction.

A single-center comparative analysis [77] investigated the impact of gastrojejunostomy orientation on DGE incidence. One hundred and twenty-one patients were divided into a horizontal group and a vertical group based on the orientation of the gastrojejunostomy. Vertical orientation was associated with a lower incidence and severity of DGE (23.9% vs. 45.3%; *p* = 0.02, and 6.5% vs. 12%; *p* = 0.006, respectively).

In conclusion, several recent studies have compared different surgical techniques and their combinations, often arriving at discordant conclusions. As of now, no consensus has been reached, and no specific technique has been proven to definitively prevent DGE.

### 3.4. Pre- and Post-Operative Nutrition

Preoperative nutrition management in pancreatic cancer patients necessitates a comprehensive understanding of the complex interplay between the disease process and nutritional status. Pancreatic cancer induces a state of metabolic dysregulation characterized by not only elevated energy expenditure but also a significant shift in substrate utilization, promoting aberrant gluconeogenesis, prioritizing hepatic glucose production even in the presence of adequate glycogen stores, and accelerating skeletal muscle protein catabolism to sustain tumorigenesis. These metabolic derangements, in concert with chronic systemic inflammation, contribute to the development of cancer cachexia, a multifactorial syndrome marked by progressive skeletal muscle wasting, adipose tissue depletion, and systemic inflammation, which significantly impairs patient outcomes and quality of life [78,79]. More precise methods to measure energy expenditure, such as indirect calorimetry, can aid in preventing malnutrition, preserving lean body mass, and improving their overall tolerance to surgery and subsequent treatments.

Moreover, pancreatic exocrine insufficiency, commonly observed in advanced PC, further exacerbates malnutrition by impairing nutrient digestion and absorption, leading to deficiencies in fat-soluble vitamins, protein, and micronutrients [80]. Consequently, addressing malnutrition and its associated metabolic perturbations before surgery is paramount to optimize patient outcomes.

When addressing preoperative nutritional support in pancreatic surgery, several key points merit consideration. Firstly, patients with adequate nutritional status typically do not require specialized interventions. However, those at low or moderate nutritional risk may benefit from oral nutritional supplements. In cases where patients face high nutritional risk or exhibit malnutrition before pancreatic surgery, aggressive nutritional support, such as enteral nutrition via tube feeding and/or supplementary or total parenteral nutrition (PN), may be recommended if oral intake targets cannot be met [81].

Furthermore, emerging evidence suggests that immunonutrition, which incorporates specific nutrients with immunomodulatory properties, holds promise in modulating the inflammatory response, enhancing immune function, and attenuating surgical stress. Nutrients such as arginine, glutamine, omega-3 fatty acids, and nucleotides have garnered particular attention for their potential to mitigate the systemic inflammatory response, reduce infectious complications, and promote tissue healing in the perioperative period [82]. A recent systematic review and meta-analysis has demonstrated that immunonutrition markedly reduces hospitalization durations and lowers the incidence of infectious complications among patients undergoing pancreaticoduodenectomy [83].

Moreover, In the contemporary landscape of pancreatic cancer treatment, an increasing proportion of patients are being administered neoadjuvant therapy as a pivotal element of their comprehensive, multimodal treatment approach. This evolving trend underscores a significant window of opportunity to integrate and implement nutritional prehabilitation strategies into the preoperative phase of care, aiming to optimize patients’ nutritional status and overall physical resilience before surgery [84].

#### Postoperative Nutrition

Postoperative nutrition management, particularly within the context of ERAS protocols, is integral to optimizing recovery and reducing complications in patients undergoing pancreatic cancer surgery. ERAS pathways emphasize early resumption of oral intake, supported by evidence-based strategies aimed at minimizing perioperative fasting, reducing surgical stress, and enhancing postoperative recovery [2].

Early oral intake, typically initiated within hours of surgery, helps maintain gut integrity, preserve muscle mass, and attenuate the catabolic response associated with surgical stress [85]. According to a recent publication by Jing et al., early oral feeding (EOF) is a safe and effective option for postoperative feeding after pancreatic surgery. The study found that EOF is superior to nasojejunal enteral nutrition in reducing the incidence of delayed gastric emptying (DGE) after surgery [53].

By facilitating early oral feeding and implementing targeted nutritional interventions, ERAS protocols not only improve patient comfort and satisfaction but also contribute to reduced hospital length of stay, decreased complication rates, and enhanced long-term outcomes following pancreatic cancer surgery.

### 3.5. Post-Operative Analgesia

Pancreatic surgery is a major abdominal procedure associated with significant postoperative pain, which, if inadequately managed, can lead to adverse outcomes [1]. Effective pain management in the postoperative setting is critical not only for patient comfort but also for optimizing recovery, as unrelieved pain can increase complications, delay mobilization, and prolong hospital stays [2]. ERAS protocols [2] emphasize the use of multimodal analgesia approaches to minimize opioid consumption while ensuring adequate pain control, thereby promoting faster recovery and early mobilization.

The ERAS guidelines [2] advocate for multimodal analgesia, which involves combining different classes of analgesic agents and techniques to target various pain pathways. The primary goal is to reduce opioid-related side effects such as nausea, vomiting, constipation, and respiratory depression while maintaining effective pain relief. This multimodal approach typically includes the use of regional anesthesia, non-opioid analgesics, and limited opioid use as a rescue medication.

#### 3.5.1. Non-Opioid Analgesics

Non-opioid analgesics are integral components of the ERAS multimodal approach to pain management [2]. These agents work synergistically with opioids or regional anesthesia techniques to provide effective pain control while reducing opioid-related adverse effects.

Paracetamol (acetaminophen), administered orally or intravenously, is recommended as a first-line analgesic due to its efficacy in managing mild-to-moderate pain and its favorable side effect profile. For optimal effectiveness, paracetamol should be administered regularly every six hours [86].

Non-steroidal anti-inflammatory drugs (NSAIDs), such as ibuprofen or ketorolac, are effective for managing inflammatory pain but should be used cautiously in patients with renal impairment or those at risk for gastrointestinal bleeding [87]. Their use is encouraged in the early postoperative period to reduce opioid requirements. The risk of NSAID-induced bleeding can be mitigated by using COX-2 inhibitors, which do not affect platelet function [88].

Additionally, gabapentin or pregabalin, which target neuropathic pain pathways, may be considered preoperatively or in the immediate postoperative period to reduce the likelihood of chronic pain and minimize opioid consumption.

#### 3.5.2. Epidural Analgesia

Thoracic epidural analgesia (TEA) provides superior pain control by blocking afferent pain signals from the abdominal region and has been shown to reduce the need for systemic opioids, particularly in cases involving large abdominal incisions [89]. A catheter delivering a continuous infusion of low-concentration local anesthetic combined with low-dose opioids enhances efficacy while minimizing unwanted side effects, such as motor block and hypotension from sympathetic block. According to ERAS guidelines [2], TEA offers excellent postoperative pain management, with additional benefits including reduced postoperative ileus through sympathetic nerve blockade, improved respiratory function by alleviating diaphragmatic splinting caused by pain, and the potential for earlier mobilization and shorter hospital stays [90,91,92].

#### 3.5.3. Transversus Abdominis Plane (TAP) Block

TAP block is a regional anesthetic technique involving the injection of local anesthetics into the neurovascular plane between the internal oblique and transversus abdominis muscles, providing somatic pain relief for the anterior abdominal wall. TAP blocks can be performed bilaterally to cover lower abdominal incisions and are typically used as an adjunct to systemic analgesics or epidural anesthesia. Recent studies suggest that combining TAP blocks with a multimodal analgesic regimen can reduce opioid consumption and enhance postoperative recovery [93]. While widely used in minimally invasive colorectal surgery, their use in pancreatic surgery is not currently recommended due to a lack of supporting studies in the literature.

#### 3.5.4. Opioid Drugs

Although ERAS protocols aim to minimize opioid use [2], opioids remain an important option for managing breakthrough pain following pancreatic surgery. When required, opioids should be used judiciously, with an emphasis on minimizing the total dose and duration of treatment.

Preferred agents include short-acting opioids such as morphine or hydromorphone, administered via IV patient-controlled analgesia (IV-PCA) or in low doses for rescue analgesia. Long-acting opioids should be avoided due to their higher risk of sedation, respiratory depression, and delayed recovery. Close monitoring for opioid-related side effects is essential, and opioid-sparing strategies should always be employed [94].

Frequent and systematic pain assessment is essential for guiding analgesia and ensuring adequate pain control in the postoperative period. ERAS guidelines [2] recommend using validated pain scoring systems, such as the Numerical Rating Scale (NRS) or Visual Analog Scale (VAS), to assess pain both at rest and during activity. Regular assessments allow for timely adjustments to analgesic regimens, ensuring effective pain management while minimizing side effects.

Inadequate pain control can lead to complications such as delayed mobilization, an increased risk of thromboembolism, impaired respiratory function (leading to atelectasis or pneumonia), and prolonged postoperative ileus due to sympathetic overactivity.

In conclusion, effective postoperative pain management is a cornerstone of the ERAS pathway for pancreatic surgery. Multimodal analgesia, centered on techniques such as thoracic epidural analgesia and non-opioid analgesics, is recommended to optimize recovery, reduce opioid-related complications, and improve patient outcomes. A tailored approach that combines regional anesthesia, systemic analgesics, and judicious opioid use promotes early mobilization and minimizes the risk of chronic pain and other postoperative complications. Continuous evaluation and adjustment of analgesic protocols are critical to ensuring a successful recovery in patients undergoing pancreatic surgery.

## 4. Conclusions

The application of the ERAS protocol in pancreatic surgery has demonstrated significant benefits, particularly in reducing morbidity, hospital stay, and postoperative complications. This review highlights the positive impact of ERAS on perioperative care, emphasizing the critical role of multidisciplinary approaches and evidence-based practices in improving patient outcomes. However, several limitations must be acknowledged. Only a limited number of ERAS recommendations are currently supported by multicenter randomized clinical trials, with a substantial portion based on retrospective case series or meta-analyses of retrospective studies. This has inevitably led to a lack of high levels of evidence or strong grades of recommendation for certain interventions.

Therefore, multicenter randomized clinical trials are still required to further validate the superiority of specific ERAS interventions, particularly in relation to potential strategies such as the use of SA, surgical drainage positioning for POPF prevention, and determining the optimal surgical reconstruction technique to mitigate DGE onset.

## Supplementary Materials

The following supporting information can be downloaded at: https://www.mdpi.com/article/10.3390/jcm13206205/s1, Table S1: A summary of the clinical trials on pancreatic surgery.
