# Supplementary material for: Enhanced Recovery After Surgery (ERAS) in Pancreatic Surgery: The Surgeon’s Point of View
Source: J Clin Med. 2024 Oct 18;13(20):6205. doi: 10.3390/jcm13206205 (PMC11508928; doi:10.3390/jcm13206205)
Supplement: Supplementary file 1 [file jcm-13-06205-s001.zip › jcm-3223486-supplementary.pdf]

Table S1. A summary of the clinical trials on pancreatic surgery.

Author and year of publication	Type of study	Study population	Endpoints	Results
Dae Wook Hwang et al. (2019) [12]	Noninferiority, single center, randomized controlled trial: patients who underwent open PD, divided into ERAS (*n* = 123) vs. conventional (*n* = 124) management. Duration: 26 months.	Patients undergoing PD with resectable cancer without metastasis or borderline malignancy. Age (ERAS vs. conventional): 63.3 ± 9.2 vs. 62.9 ± 9.2 Male (%): 72 (58.5) vs. 81 (65.3)	Primary: incidence of overall morbidity until postoperative 3 months. Secondary: in-hospital or 30-day mortality, LOS, nutritional status, hospital costs.	ERAS protocol was not inferior to conventional management regarding the overall morbidity until 3 months after surgery (64 vs. 68 patients), mortality (none), median postoperative LOS (both 11 days) and costs.
Julie Perinel et al. (2019) [13]	Prospective controlled before/after study: patients undergoing PD, divided into ERAS program (*n* = 97) vs. traditional care (*n* = 75). Duration: 24 months.	Patients undergoing elective pancreatic resection. Age (ERAS vs. conventional): 60.4 ± 13.5 vs. 62.3 ± 13.5 Male (%): 54 (56) vs. 37 (49)	To evaluate the impact of the implementation of the ERAS program on postoperative outcomes and to report the compliance to the ERAS program.	Implementation of ERAS program was safe and effective with high compliance rate (OR 1.34; 95% CI 1.18–1.53). LOS was significantly reduced (HR 1.61; 95% CI 1.07–2.44).
Muhammer Ergenc et al. (2021) [14]	Prospective randomized controlled clinical trial: patients who underwent open PD, divided into ERAS (*n* = 18) vs. conventional (*n* = 20) management. Duration: 34 months.	Patients undergoing elective open pancreatic cancer surgery. Age (ERAS vs. conventional): 51 (36 – 76) vs. 64 (28 – 85) Male (%): 9 (50) vs. 13 (65)	To determine whether there was a difference between the two groups in terms of post- operative complications, length of hospital stay, and incidence of readmission rates.	ERAS protocol provides a minimal decrease in the total complication rate and has no effect on severe complications such as abdominal infection, LOS, DGE, and overall mortality.
Harish Lavu et al. (2019) [15]	Prospective randomized controlled trial: patients who underwent open PD, divided into Whipple accelerated recovery pathway (WARP) (*n* = 37) vs. conventional (*n* = 39) management. Duration: 26 months.	Patients undergoing elective pancreatic surgery. Age (WARP vs. conventional): 65.8 ± 9.6 vs. 65.0 ± 9.3 Male (%): 20 (54.1) vs. 18 (46.2)	Primary: percentage of patients discharged by POD 5. Secondary: complications, readmissions, time to adjuvant therapy commencement, and total hospital charges and cost.	WARP effectively facilitated recovery after PD: the rate of POD 5 discharge was 74.3% (26 of 35) vs. 14.3% (5 of 35) in the control arm ($p < 0.001$). Hospital LOS, time to adjuvant therapy, hospital cost was all reduced by the WARP.
Xiaxing Deng et al. (2017) [16]	Randomized controlled clinical trial: patients who underwent open PD, divided into ERAS (*n* = 76) vs. conventional (*n* = 83) management. Duration: 24 months	Patients undergoing PD with resectable cancer. Age (ERAS vs. conventional): 54.5 ± 12.7 vs. 51.3 ± 15 Male (%): 46 (60.5) vs. 46 (55.4)	To investigate the feasibility and safety of implementing the ERAS protocol in patients undergoing PD. The outcome measured included postoperative complications, LOS and 90-day mortality rates.	ERAS protocol was safe and beneficial, reducing LOS without increasing the incidence of complications such as POPF (39 vs. 36, ERAS vs. conventional, $p = 0.52$), post-operative hemorrhage (6 vs. 5, $p = 0.5$) and mortality (none).
Maria Kapritsou et al. (2020) [17]	Prospective randomized controlled clinical trial: patients who underwent	Patients undergoing PD with resectable cancer.	To comparatively evaluate ERAS and conventional protocols in patients undergoing PD regarding pain intensity,	Decreased postoperative pain ($p = .002$) was observed in ERAS patients.

	open PD, divided into ERAS (*n* = 44) vs. conventional (*n* = 41) management. Duration: 34 months.	Age (ERAS vs. conventional): 60.5 ± 11.7 vs. 64.8 ± 11.9 Male (%): 29 (65.9) vs. 23 (56.1)	emotional response, and stress biomarker (ACTH, cortisol) levels.	The type of protocol does not appear to have a significant effect on hormone levels nor on self-reported stress/emotional responses.
Kosei Takagi et al. (2019) [18]	Prospective randomized clinical trial: patients who underwent open PD, divided into ERAS (*n* = 37) vs. conventional (*n* = 37) management. Duration: 34 months.	Patients undergoing elective PD. Age (ERAS vs. conventional): 69.8 ± 9.7 vs. 66.8 ± 9.3 Male (%): 20 (54) vs. 20 (54).	Primary: postoperative LOS. Secondary: postoperative complications, compliance with ERAS protocol, readmission rates, mortality rates, medical costs.	In the ERAS group mean LOS was much shorter (20.1 ± 5.4 vs. 26.9 ± 13.5 days, $p < 0.001$), percentage of complications 32.4% vs. 56.8%, $p = 0.034$) and readmissions (0% vs. 8.1%, $p = 0.038$) were significantly lower. Total medical cost was also lower ($p = 0.085$).

Abbreviations: PD, pancreatoduodenectomy; LOS, length of stay; OR, odds ratio; HR, hazard ratio; POD, post operative discharge; POPF, post operative pancreatic fistula; DGE, delayed gastric emptying.
